# LETTER TO THE EDITOR An Unusual Burn Caused by Heated Car Seat

**Published:** 2010-04-08

**Authors:** Celalettin Sever, Yalcin Kulahci, Fatih Uygur, Sinan Oksuz

**Affiliations:** Department of Plastic and Reconstructive Surgery and Burn Unit, Gülhane Military Medical Academy and Medical Faculty, Haydarpasa Training Hospital, Istanbul, Turkey

Dear Sir,

We would like to report a case of a 38-year-old man who suffered second- and third-degree burns measuring approximately 3 × 12 cm to his right perianal region as the result of contact with a faulty car seat heater (Fig 1). The patient was a backseat passenger who was paraplegic with underlying diabetes mellitus. The burn area was treated conservatively over a 5-week period without surgery. To our knowledge, despite its widespread use, only a few burn cases have been reported in the literature.^1^^-^3

Car seat technology has added numerous complex features to modern cars, including seat heaters, seat coolers, and computerized controls to adjust seat position. Once considered a luxury item, electric heated car seats are now commonplace and have been known to malfunction and become dangerously hot, which may cause third-degree burns. The surface temperature at the heated car seat may reach 120°F. This temperature may cause localized deep and even life-threatening burns within 10 minutes.^1^ Therefore, this injury is a major potential risk for patients with sensory deficits such as paraplegia, diabetes, vascular disease, stroke, and mental or physical disabilities. Small children may be unaware that the car seat heater is switched on and may also get injured. These burns are preventable and, therefore, some basic measures may reduce the incidence of accidental burn injury due to heated car seats.

1. The seat heaters on every car should be thoroughly tested to increase their safety and have a maximum temperature setting.
2. The car manufacturers should consider posting warnings in their owners' manuals highlighting the potential danger of burns due to heated seats.

We hope that this case report will succeed in raising awareness of this problem.

## Figures and Tables

**Figure 1:**
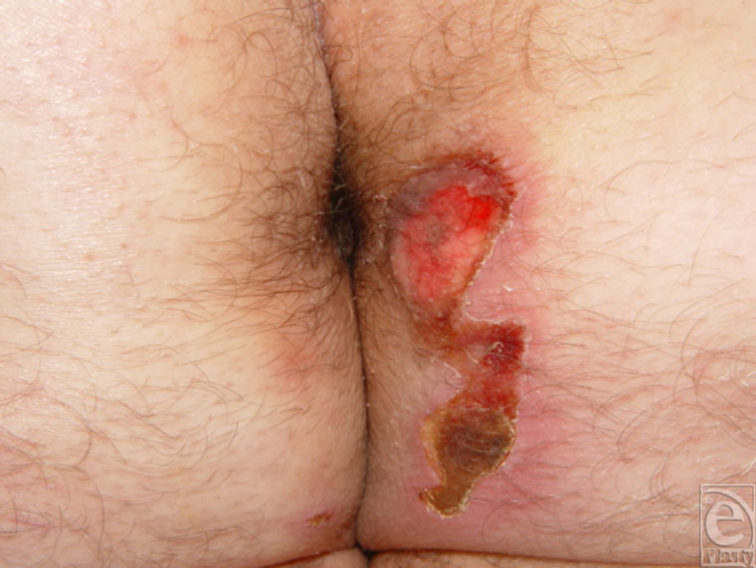
Contact burn affecting the perianal region of the patient.
